# Supplementary material for: Chitosan-Coated PLGA Nanoparticles Encapsulating Triamcinolone Acetonide as a Potential Candidate for Sustained Ocular Drug Delivery
Source: Pharmaceutics. 2021 Sep 30;13(10):1590. doi: 10.3390/pharmaceutics13101590 (PMC8541202; doi:10.3390/pharmaceutics13101590)
Supplement: Supplementary file 1 [file pharmaceutics-13-01590-s001.zip › pharmaceutics-1340841-supplementary.pdf]

pharmaceutics

MDPI

Supplementary Materials: Chitosan-Coated PLGA Nanoparticles Encapsulating Triamcinolone Acetonide as a Potential Candidate for Sustained Ocular Drug Delivery

Madhuri Dandamudi, Peter McLoughlin, Gautam Behl, Sweta Rani, Lee Coffey, Anuj Chauhan, David Kent and Laurence Fitzhenry

Table S1. Design of experiments generated by Box-Behnken design statistical design in Minitab software.

Run Order	PLGA (mg/mL)	Poloxamer % (*w*/*v*)	Chitosan % (*w*/*v*)
1	4	2	2
2	4.5	1.5	2
3	3.5	1.5	2
4	3.5	1.5	2
5	3.5	2	1.5
6	4	1	1
7	3.5	1.5	1
8	4.5	1	1.5
9	4.5	2	1.5
10	3.5	1.5	1
11	4.5	1	1.5
12	4	1.5	1.5
13	4.5	2	1.5
14	3.5	1	1.5
15	4.5	1.5	1
16	4	2	1
17	4	2	1
18	3.5	1	1.5
19	4	1.5	1.5
20	4	1	2
21	4	1.5	1.5
22	4	2	2
23	4	1	1
24	4.5	1.5	2
25	3.5	2	1.5
26	4	1.5	1.5
27	4	1.5	1.5
28	4	1.5	1.5
29	4	1	2
30	4.5	1.5	1

Table S2. Investigated values of responses for the prepared nanoparticles using Box-Behnken design.

Run Order	PLGA (mg/mL)	Poloxamer % (*w*/*v*)	Chitosan % (*w*/*v*)	Particle size (nm)	PDI	Zeta Potential (mV)	% Encapsulation Efficiency
1	4.0	2.0	2.0	340.00	0.0910	10.50	34.07
2	4.5	1.5	2.0	420.00	0.0640	28.60	34.34
3	3.5	1.5	2.0	333.00	0.0420	37.80	36.56
4	3.5	1.5	2.0	410.00	0.4700	31.20	36.90
5	3.5	2.0	1.5	569.00	0.1021	-46.40	51.92
6	4.0	1.0	1.0	340.00	0.1180	52.54	48.95
7	3.5	1.5	1.0	819.00	0.1200	46.90	28.79
8	4.5	1.0	1.5	414.00	0.1450	22.70	53.40
9	4.5	2.0	1.5	337.00	0.1170	-42.70	50.95
10	3.5	1.5	1.0	474.36	0.1710	29.40	33.48
11	4.5	1.0	1.5	464.00	0.0970	25.60	56.38
12	4.0	1.5	1.5	349.00	0.1140	20.40	53.43
13	4.5	2.0	1.5	380.00	0.2440	-38.20	52.15
14	3.5	1.0	1.5	286.90	0.1090	19.60	57.79
15	4.5	1.5	1.0	410.00	0.1570	36.89	30.94
16	4.0	2.0	1.0	350.00	0.2360	-35.60	37.46
17	4.0	2.0	1.0	390.00	0.1210	-28.40	34.37
18	3.5	1.0	1.5	383.00	0.0710	33.70	52.56
19	4.0	1.5	1.5	465.00	0.1180	25.52	50.67
20	4.0	1.0	2.0	480.00	0.2830	57.70	33.41
21	4.0	1.5	1.5	390.00	0.0840	31.50	56.26
22	4.0	2.0	2.0	370.70	0.2203	18.20	31.98
23	4.0	1.0	1.0	525.00	0.1340	48.80	49.51
24	4.5	1.5	2.0	372.77	0.0884	18.90	31.16
25	3.5	2.0	1.5	420.00	0.1120	-33.20	35.28
26	4.0	1.5	1.5	268.70	0.1870	29.30	41.07
27	4.0	1.5	1.5	441.00	0.0450	30.60	39.09
28	4.0	1.5	1.5	471.00	0.4120	28.60	38.21
29	4.0	1.0	2.0	427.00	0.1210	33.40	27.62
30	4.5	1.5	1.0	361.00	0.1650	42.70	30.38

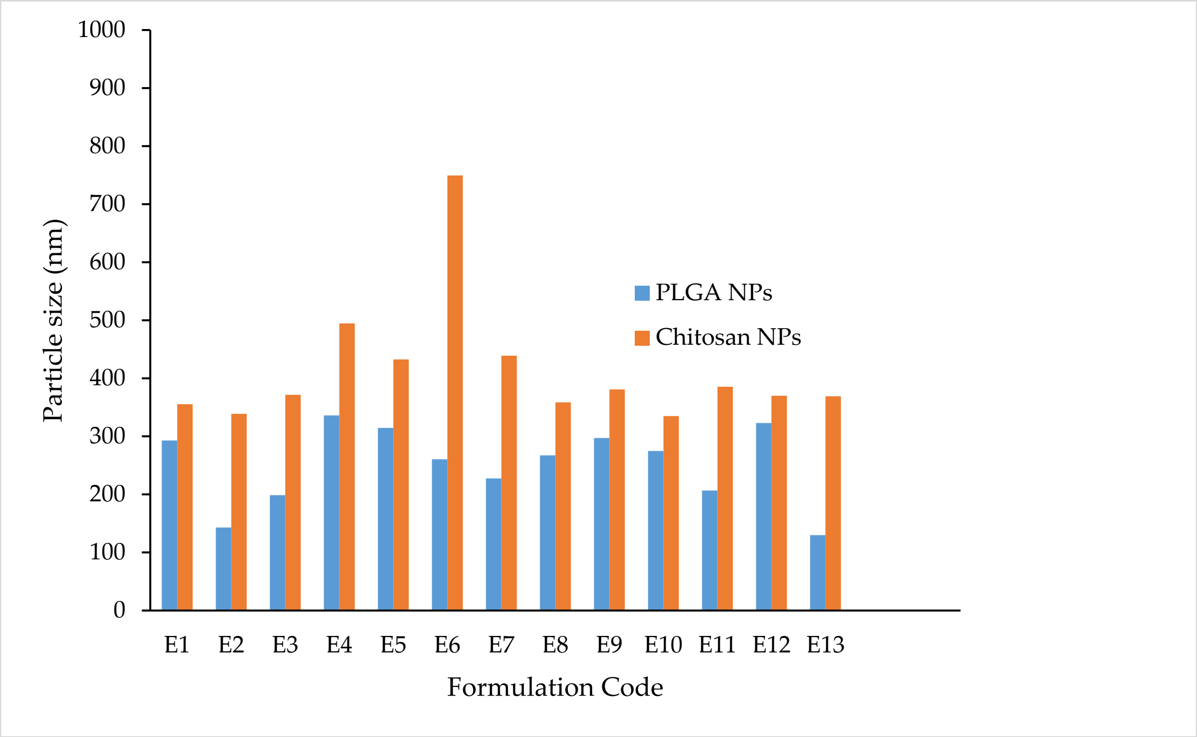

Figure S1. Comparison of particle size (nm) of PLGA NPs before and after coating with chitosan.

Table S3. Predicted experimental conditions for low particle size and high encapsulation efficiency for CS-PLGA nanoparticles.

**Response**	**Goal**	**Lower**	**Target**	**Upper**	**Weight**	**Importance**
% Encapsulation Efficiency	Maximum	27.62	57.79		1	1
Particle size (nm)	Minimum		257.70	819	1	1
**Solution**	**PLGA (mg/mL)**	**Poloxamer % (*w/v*)**	**Chitosan % (*w/v*)**	**%Encapsulation Efficiency Fit**	**Particle size (nm) Fit**	**Composite Desirability**
1	3.70202	1	1.51515	55.1770	388.462	0.837021
**Response**		**Fit**	**Standard Error of the Fit**	**95% CI**		**95% PI**
% Encapsulation Efficiency		55.18	2.97	(48.98, 61.37)		(40.85, 69.50)
Particle size (nm)		388.5	46.7	(291.1, 485.8)		(163.2, 613.7)

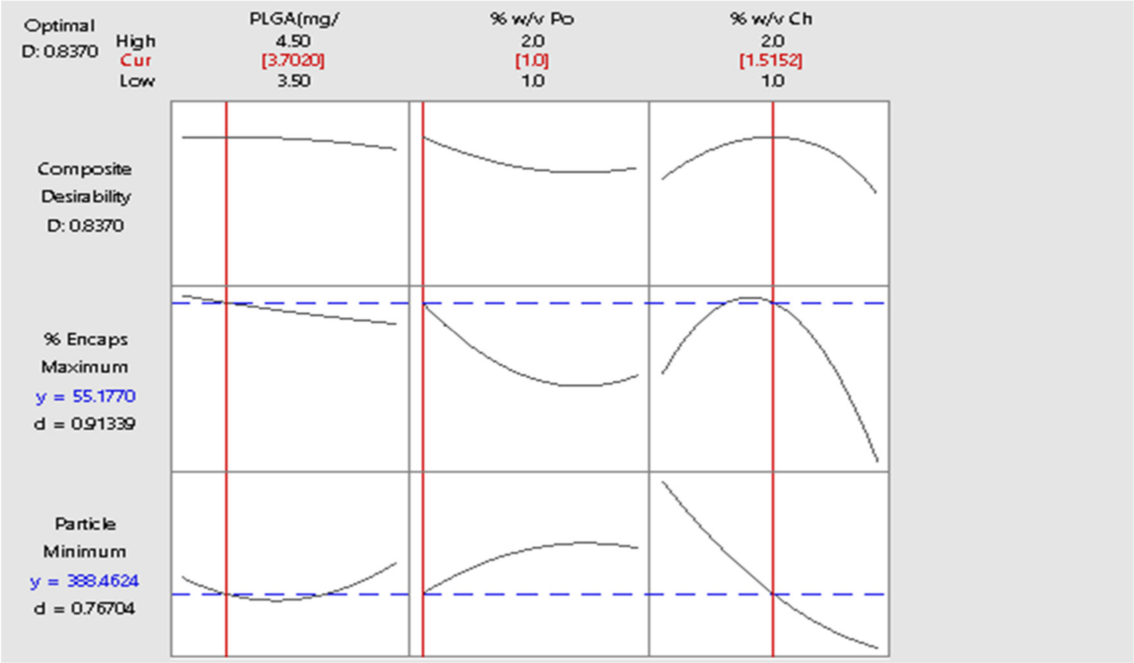

Figure S2. Response optimization parameters and predicted experimental conditions for low particle size and high encapsulation efficiency for CS-PLGA nanoparticles.

Table S4. Predicted experimental conditions for low particle size and + 25 mV zeta potential efficiency for CS-PLGA nanoparticles.

Response	Goal	Lower	Target	Upper	Weight	Importance
Zeta Potential (mV)	+25	−46.4	+25	+48	1	1
Particle Size (nm)	Minimum		257.70	819	1	1

Solution	PLGA (mg/mL)	Poloxamer % (*w/v*)	Chitosan % (*w/v*)	Zeta potential (mV) Fit	Particle size (nm) Fit	Composite Desirability
1	4.49841	1	1.31815	25.0000	388.462	1.00000

Response	Fit	Standard Error of the Fit	95% CI	95% PI
Zeta Potential (mV)	25.00	8.16	(7.97, 42.03)	(−6.86, 56.86)
Particle Size (nm)	388.5	46.7	(291.1, 485.8)	(163.2, 613.7)

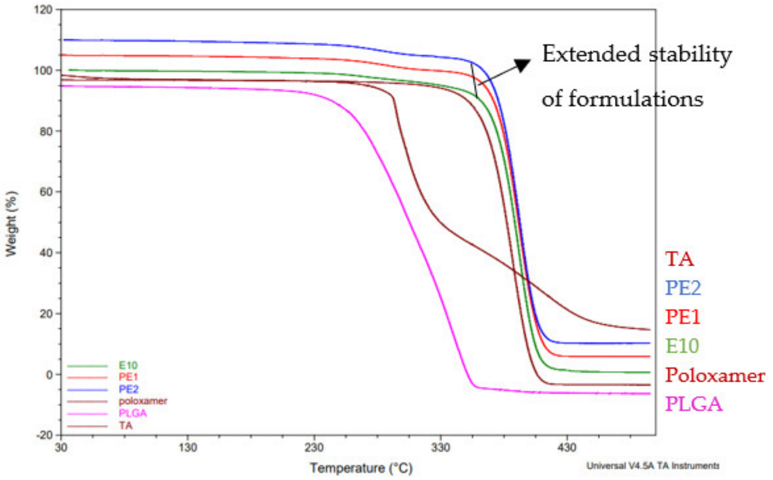

Figure S3. Thermal analysis of individual components and PLGA NPs (PE1, PE2 and E10).

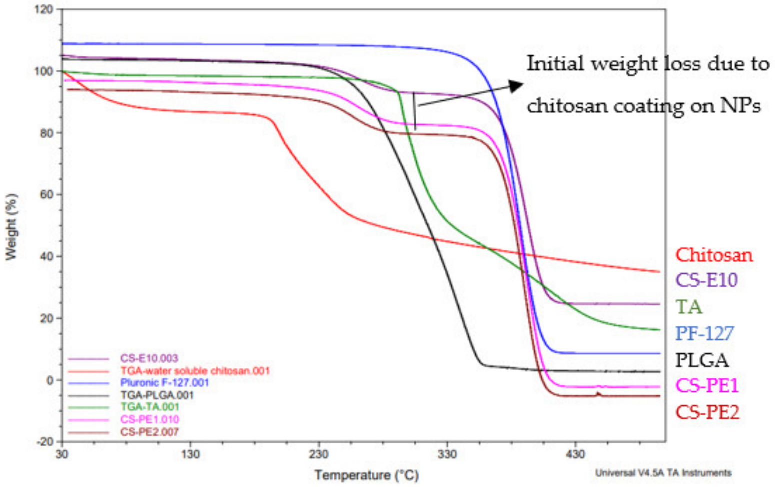

Figure S4. Thermal analysis of chitosan-coated NPs (CS-E10, CS-PE1 and CS-PE2) and the individual components using TGA.

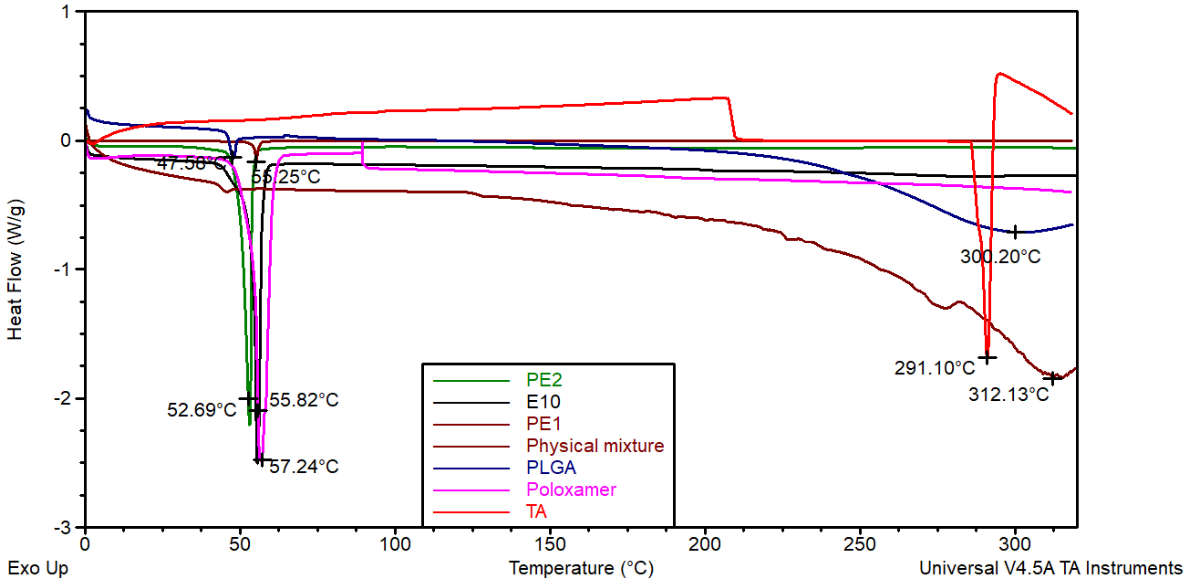

Figure S5. DSC thermograms of components and PLGA NPs (E10, PE1 and PE2).
